# Faulty screen time measures hamper national policies: here is a way to address it

**DOI:** 10.3389/fpsyg.2023.1243396

**Published:** 2023-07-27

**Authors:** Natalia I. Kucirkova, Sonia Livingstone, Jenny S. Radesky

**Affiliations:** ^1^The Open University, Faculty of Education, Milton Keynes, United Kingdom; ^2^Learning Environment Centre, University of Stavanger, Stavanger, Norway; ^3^London School of Economics and Political Science, London, United Kingdom; ^4^Department of Pediatrics, University of Michigan Medical School, Ann Arbor, MI, United States

**Keywords:** screen time, early development, guidelines and recommendations, psychology science, technologies

Following widespread reliance on online interactions during COVID-19, Western governments are strategizing and launching new plans for children's use of screens. It is healthy to debate, for example, the ethics of artificial intelligence (AI) or K12 learning loss in relation to children's “screen time” with social media or educational technologies. However, current screen time debates obscure a central insight from research on children's digital media: namely that precise and detailed considerations of the content, context and children's characteristics - as well as the underlying design infrastructure of digital technologies that shape children's opportunities—are essential for operationally relevant and practical guidelines for the public.

In this Opinion article, we reveal methodological shortcomings of screen time measures deriving from the disconnect between the affordances of current and older digital media. We explain how different interpretations of the evidence base led to screen time guidelines in around the world that are, in turn, disconnected from family experiences. To provide a useful proxy to guide national policies, we recommend a measurement of digital media engagement that takes into account attitudes and practices; content and context; short bursts as well as the complexity of children's overall media usage (Barr et al., 2020) and media's evolving design affordances.

## Traditional measures

Since the first comprehensive large-scale psychology studies concerning the impact of television on children's social and cognitive learning (led by Schramm, 1965 in USA and Himmelweit et al., 1958 in the UK), the screen time debate in early childhood has been ongoing in some form and with intense public and empirical examination in many disciplines, albeit escalating in the 1990's with the multiplication of screens in the home (Livingstone, 2021). The cross-stakeholder engagement and vibrant research interest across disciplinary fields arises from the significant presence that screens have in children's lives: national and international studies show increasing daily use of screens by increasingly younger children, a trend that intensified during national lockdowns in 2020-2022, as documented across countries (e.g., in Germany by Schmidt et al., 2020 and Aguilar-Farias et al., 2021 in Chile).

Traditional measures based on parent-reported duration of media use (e.g., “On a typical weekday, how much time does your child spend watching TV?”) are ill-equipped to measure children's modern digital experiences. Decades ago, when parents could recall how many TV episodes or DVDs their child watched, recall of duration was a more reasonable metric and was easy to add to any large-scale survey of child wellbeing. Parents may have been asked to additionally report the content watched by their child, which was easier for researchers to categorize (i.e., as educational or entertainment) as a limited number of TV shows were produced and aired each day. Screen duration variables were also easy to plug into regression models as unidimensional continuous or categorical predictors (see recent meta-analyses of screen time research: Madigan et al., 2019; Eirich et al., 2022).

Traditional methodologies also included use of time use diaries, which provided more insight into how screen use fitted into the larger context of daily routines and family activities. However, parents now have variable involvement in their children's use of handheld technology (Domoff et al., 2019), and cannot easily watch over their shoulders. While they may still know how long the children spend online, they are less sure of the content their child accesses on different platforms, or the nature of their engagement, especially for busy or overworked parents. In addition, time use diaries typically capture discrete units of time spent on different activities, but are less suited to measuring multitasking, switching between digital activities within the same period of “screen time,” fragmentation of other activities with technology, or, crucially, the nature of child-screen interaction (e.g., purchases, persuasive design, risky content, see Kidron et al., 2018). As a result, the accuracy of time use diary methods is undermined.

Psychological science has traditionally approached children's use of digital technologies from the perspectives of learning domains (e.g., language gains), milestones of development (e.g., fine motor development at three years of age), or testing hypotheses about, for example, socialization or emotional development. Laboratory-based experiments have generated insights into what children at different ages can learn from screen-based media (e.g., novel words, visual problem-solving) compared with interactions with a caregiver (e.g., Arnold et al., 2021). Lab-based experiments have utilized bespoke videos/interactive media (i.e., created to test a particular hypothesis, e.g., Escudero et al., 2021; Korat et al., 2022) or commercially-available videos/apps (e.g. Dore et al., 2019), but generally have not been able to evaluate children's responses to the vast range of digital products used in their home and school environments.

Finally, traditional approaches used in children's media research have been biased in terms of populations studied and perspectives included. With notable exceptions of government-run surveys or research groups that focus on underrepresented groups (see e.g., Mendelsohn et al., 2008), the typical approach of recruiting families through university registries, local organizations, or social networks has tended to recruit a predominantly White, highly educated and motivated population who likely have distinct attitudes, anxieties, and practices around media (Alper et al., 2016). Limited research with families from marginalized races/ethnicities may be driven by lack of trust in academic research and government-funded institutions due to prior transgressions and privacy concerns (Smirnoff et al., 2018). Thus, screen time research describes behaviors of predominantly White middle class families, without sufficiently examining the rich differences in how media use is interwoven into different families' lives.

## Screen time guidelines and national policies

In recent decades, guidelines have been produced by child health authorities in many countries, such as the American Academy of Pediatrics (AAP), UK Royal College of Pediatrics and Child Health, Australian Government Department of Health (2018), and Canadian Paediatric Society Digital Health Task Force (2019), as well as the World Health Organization (WHO). The more restrictive guidelines recommend no screen use for children under two years of age and not more than one hour a day of sedentary screen time for toddlers (children between the ages of 2–5). With the goal of being evidence-based, these health authorities have reviewed published evidence and interpreted findings in relation to local clinical experience, socioeconomic conditions, and parenting culture. Author 3 led one of the national guidelines and therefore has experience in critically reviewing how the published literature is translated into guidelines. Collectively, as authors we recognize three sources of systematic bias in these guidelines.

First, guidelines are only as rigorous as the body of evidence upon which they are based. Therefore, the inherent limitations of the “screen time” paradigm prevalent in published literature described above are then transferred into guidelines. For example, if studies do not distinguish between types of technologies, platforms, or the nature of context-child-computer interaction, these important concepts will not be included in the guidelines. If interventional research focuses only on the problems associated with screen time overall, then policies tend to prioritize restricting children's digital engagement (e.g., recent U.S. state laws requiring parental consent for adolescent social media usage) rather than changing underlying digital structures that influence child online risk (e.g., data profiling and algorithmic recommendations) or, indeed, promoting digital opportunities.

Second, the guidelines are based on certain *assumptions* that researchers of diverse disciplines and traditions bring with them. Assumptions about how children learn are different in education and psychology, for example, and while in many studies the differences are reconciled through interdisciplinary approaches toward individual and socio-cultural approaches, this is rarely the case in national guidelines. For example, health research tends to focus on health outcomes and clinical settings, with assumptions that it is best to optimize health but with the risk of ignoring socio/technical/cultural issues; educational research tends to focus on measurable educational outcomes with the risk of ignoring less easily measurable informal learning.

Third, population bias (and the overall whiteness of researchers) is an issue in the research underpinning national guidelines. The universal nature of national guidelines is in tension with the highly individualized and idiosyncratic nature of technologies designed for personal and personalized use. Due to the myriad ways families use media, it is more important to focus on “healthy” or human-centered default settings so that it is easiest for all users to get the most out of their specific technology use, rather than trying to force a top-down approach that recommends normative behaviors. The population bias is a systematic bias and relates to the limited input and representation of the lived experiences of populations and their unique contexts of technology use.

Scholars have pointed out discrepancies between guidelines, which may be explained by different interpretation of an evidence base characterized by variable rigor and quality (see Stiglic and Viner, 2019; Odgers and Jensen, 2020). For example, WHO's more restrictive guidelines differ from the Royal College of Pediatrics and Child Health (RCPCH) in the United Kingdom that recommends parents make decisions based on their own judgement of the child's situation and needs and only recommends avoiding screen use one hour before bedtime (Royal College of Paediatrics Child Health, 2019). WHO's and AAP's harm-prevention approach has been criticized for being based on a restriction- and reduction-oriented medical model that disregards the socio-cultural and civic benefits of children's use of screens (Livingstone and Blum-Ross, 2020). A recent overview of international screen time guidelines details the ways in which global screen measures fail to recognize the varying needs and vulnerabilities of children and their digital rights (Straker et al., 2023) and points to the need to measure efficiently the various kinds of interactions children have with digital media. With more nuanced research that recognizes the diverse contexts, contents and types of technology use, there will be less of scientific contestation and a greater opportunity to evolve unified guidelines across different health authorities.

In sum, today's technologies are multifunctional and multifaceted and the impact of their use on families' needs should take into account their new roles and affordances for family interactions. An updated evidence base that is informed by communities, precise in its multi-level measurement, and reflective of its underlying assumptions is needed to inform future rounds of public guidance on children's use of screens. Comprehensive yet detailed media assessments (e.g., Barr et al., 2020) open possibilities for a more precise language in discussing children's screen use in the age of AI.

## Author contributions

All authors listed have made a substantial, direct, and intellectual contribution to the work and approved it for publication.
